# Hydrogel–Nanolipid Formulations for the Complex Anti-Inflammatory and Antimicrobial Therapy of Periodontitis

**DOI:** 10.3390/pharmaceutics17050620

**Published:** 2025-05-07

**Authors:** Rabia Ashfaq, Nóra Tóth, Anita Kovács, Szilvia Berkó, Gábor Katona, Rita Ambrus, Tamás Ferenc Polgár, Mária Szécsényi, Katalin Burián, Mária Budai-Szűcs

**Affiliations:** 1 Institute of Pharmaceutical Technology and Regulatory Affairs, Faculty of Pharmacy, University of Szeged, H-6720 Szeged, Hungary; rabia.ashfaq@szte.hu (R.A.); gasparne.kovacs.anita@szte.hu (A.K.); berko.szilvia@szte.hu (S.B.); katona.gabor@szte.hu (G.K.); ambrus.rita@szte.hu (R.A.); 2 Core Facility, HUN-REN Biological Research Centre, H-6726 Szeged, Hungary; polgar.tamas@brc.hu; 3 Theoretical Medicine Doctoral School, University of Szeged, H-6722 Szeged, Hungary; 4 Department of Medical Microbiology, University of Szeged, H-6720 Szeged, Hungary; szecsenyi.maria@med.u-szeged.hu (M.S.);

**Keywords:** clove oil, HPMC, hydrogels, meloxicam, nanostructured lipid carrier, sodium hyaluronate, zinc hyaluronate

## Abstract

**Objectives**: This study aimed to develop and evaluate nanostructured lipid carriers (NLCs) loaded with meloxicam (Melox) and a therapeutic antibacterial and anti-inflammatory liquid lipid, clove oil (CO) for periodontitis treatment, a complex inflammatory condition necessitating advanced drug delivery systems. The NLC–Melox formulation was integrated into three hydrogels, hypromellose (HPMC), zinc hyaluronate (ZnHA), and sodium hyaluronate (NaHA), to conduct a comparative analysis focusing on enhanced localized drug delivery, improved mucoadhesion, prolonged retention, and significant therapeutic outcomes. **Methods:** NLC–Melox was prepared by homogenization and characterized by dynamic light scattering (DLS). Subsequently, NLC–Melox-loaded gels were subjected to transmission electron microscopy (TEM), differential scanning calorimetry (DSC), X-ray diffraction (XRD), Raman spectroscopy, and rheological analysis. In vitro drug release, anti-inflammatory activity (BSA denaturation assay), and antibacterial efficacy (MIC, MBC) were investigated to assess therapeutic potential. **Results:** DLS revealed a particle size of 183 nm with a polydispersity index of 0.26, indicating homogeneity. TEM confirmed consistent morphology and uniform nanoparticle distribution. DSC and XRD demonstrated the amorphous nature of Melox, enhancing solubility and stability. Spectroscopy confirmed no chemical interactions between components. Rheological studies identified ZnHA as the most mucoadhesive and structurally stable gel. In vitro release studies showed sustained drug release over 24 h. Melox and CO-loaded formulations demonstrated significant anti-inflammatory activity and notable antibacterial efficacy due to the antibacterial oil. **Conclusions:** The study highlighted the potential of NLC-based mucoadhesive hydrogels as an effective strategy for periodontitis treatment. The formulation offered improved drug solubility, therapeutic efficacy, mucoadhesivity, and prolonged delivery, making it a promising candidate for localized therapy.

## 1. Introduction

Periodontitis, a prevalent inflammatory condition of the periodontal tissues, poses significant challenges in clinical management due to its complex etiology involving microbial infection and inflammatory responses [1]. Treatment options for inflammatory periodontitis include mechanical extractions and the administration of antibacterial medications into the intraperiodontal pocket. Traditional treatments often fall short in addressing the multifaceted nature of the disease and entail the development of advanced drug delivery systems to enhance therapeutic efficacy and clinical outcomes. Thus, syringeable semisolid bioadhesive networks can be designed to have three main characteristics: requisitory flow property that makes it easy to inject with a syringe, mucoadhesive properties that guarantee long-term retention in the periodontal pocket, and continuous release of the therapeutic drug in the pocket [2].

Numerous studies have explored targeted therapeutic approaches for periodontal tissue healing and regeneration. For example, researchers prepared a chlorhexidine-loaded polymeric nanoparticle system integrated into a thermosensitive gel, which demonstrated improved drug retention, prolonged antimicrobial release, and lower cytotoxicity [3]. In a novel approach, minocycline-loaded mesoporous bioactive glass nanoparticles were incorporated into a photo-crosslinkable hydrogel, offering a multifunctional platform with antibacterial, anti-inflammatory, and osteogenic features for guided bone regeneration in periodontal disease therapy [4]. Furthermore, a mucoadhesive trilayer film with a solubilized eggshell membrane was found to enhance wound healing and deliver antimicrobial agents, leading to faster recovery of oral mucosal lesions [5]. Likewise, a piperine-loaded in situ gel showed extended retention in periodontal pockets, sustained drug release, and marked anti-inflammatory effects, resulting in significant clinical improvement in periodontal disease conditions [6].

Therapeutic outcomes by traditional nonsteroidal anti-inflammatory drugs (NSAIDs) are associated with blocking cyclooxygenase-2 (COX-2), whereas these drugs may also inhibit gastroprotective COX-1 isoform and trigger the side effects [7]. COX-2 selective inhibitors are associated with a lower incidence of adverse gastric events due to their limited inhibition of the protective COX-1 enzyme and delivering equivalent anti-inflammatory benefits through the targeted suppression of COX-2 [8]. Hence, a selective COX-2 inhibitor, which is considered safer compared to classical NSAIDs, is an appropriate choice for the treatment.

Meloxicam (Melox), a highly potent NSAID and selective COX-2 inhibitor, is renowned for its significant analgesic and anti-inflammatory properties [9]. These attributes make it an effective candidate for managing the pain and inflammation characteristic of periodontitis.

Clove oil (CO), containing eugenol as a major component, is traditionally used for its potent analgesic, antioxidant, anti-inflammatory, and antibacterial properties [10]. Its role as a liquid lipid helped stabilize the formulation and contributed to the antibacterial aspect of the treatment, potentially addressing oral infections more effectively. As reported by Mahadlek et al., the gel formulated with 2% xanthan gum, 20% zinc oxide (ZnO), and varying concentrations of CO or eugenol exhibited improved antibacterial efficacy with higher amounts of CO and eugenol [11].

Nanostructured lipid carriers (NLCs) have emerged as a promising strategy in drug delivery due to their ability to improve solubility, carrier stability, and the controlled release of therapeutic agents [12].

Khurana et al. published a study demonstrating the superior physical stability of Melox loaded into NLCs compared to nanoemulsions and solid lipid nanoparticles. This enhanced stability significantly minimized drug leakage, reinforcing the potential of NLCs as a reliable drug delivery system [13]. Moreover, the authors highlighted that Melox-loaded NLCs offer a promising approach for local drug delivery. When incorporated into a gel formulation, NLCs not only ensure effective localized delivery with minimal side effects but also exhibit the capacity to form a drug reservoir, enabling drug release into deeper tissues [14]. On the other hand, researchers indicated that CO-loaded NLCs are physically stable and demonstrate potent antioxidant activity, along with notable protective effects on fibroblasts. Both free CO and its NLC formulation showed strong free radical scavenging capacity. However, only the NLC-based system conferred effective protection to fibroblast cells under oxidative stress, underscoring its enhanced cytoprotective potential [15]. Another previously published investigation asserted that NLC incorporation enhances the solubility and stability of CO [16]. Although numerous studies have examined the incorporation of either Melox or CO into NLCs individually, no published research has yet explored their combined integration within a single NLC system.

Hence, this study explored a nanosizing approach to incorporate an effective anti-inflammatory drug (Melox) and therapeutic liquid lipid (CO), aiming to enhance both the stability and efficacy of the drug delivery system.

In the case of oral dosage forms, a key limitation of traditional delivery systems lies in their short retention time within the oral cavity, as they are rapidly displaced by salivary flow and mechanical actions of the tongue. To address mucosal pathologies effectively, an optimal therapeutic approach involves the use of mucoadhesive formulations that are designed to adhere firmly to the mucosal tissues, prolonging their residence at the target site. This extended retention enhances localized drug delivery to the affected area and establishes a sustained protective barrier, thereby improving therapeutic outcomes, while shielding damaged or inflamed tissues [17].

Generally, NLC dispersion possesses low viscosity and mucoadhesivity to stay at the target site for prolonged drug release purposes. Hence, optimizing a nanoparticle-loaded gelling system is crucial for keeping the nanoformulation intact for extended release. The current study aimed to analyze different polymeric systems loaded with Melox nanoparticles based on physicochemical and biomedical properties such as gelling characteristics, controlled release, antibacterial, and anti-inflammatory.

Hypromellose (HPMC) is a carbohydrate polymer known for its gel reversibility in aqueous solutions with temperature changes. Owing to its high swellability and thermal gelation properties, HPMC has emerged as a critical carrier material in drug delivery systems, particularly for controlled drug release applications. Its ability to form stable gels under varying thermal conditions makes it an ideal candidate for designing advanced drug delivery platforms, ensuring sustained and controlled release of therapeutic agents [18].

Hyaluronic acid (HA), a naturally occurring, widely recognized biodegradable, biocompatible, non-toxic, viscoelastic linear anionic polysaccharide, is a key component of the extracellular matrix in connective tissues. Structurally, HA consists of two disaccharide units (N-acetyl-d-glucosamine and d-glucuronic acid) polymerized into large macromolecules containing over 30,000 repeating units [19].

Under physiological conditions, HA exists primarily as a sodium salt, sodium hyaluronate (NaHA), and, in conjugation with zinc, zinc hyaluronate (ZnHA), making it highly soluble and functional in biological systems [20,21]. In a clinical trial, the application of NaHA gel in addition to amino acids alongside subgingival debridement led to a statistically significant decrease in bleeding and probing depth [22]. Furthermore, it accelerated the wound-healing process of soft oral tissues, promoted angiogenesis, reduced healing time, and prevented post-operative infections [23]. Meanwhile, the combination of HA and zinc in ZnHA gels offers dual therapeutic benefits: HA promotes cell proliferation, tissue regeneration, and pro-inflammatory cytokine production [24], while zinc ions exert antimicrobial effects, addressing infections and accelerating the healing of periodontal tissues [25].

These forms of HA also offer unique properties of enhanced mucoadhesion, making them suitable for sustained drug delivery in local applications and presenting a superior alternative to conventional nanotherapeutic approaches.

Altogether, the objective of this research was to fabricate and comprehensively evaluate nanolipid carriers encapsulating the drug (NLC–Melox), which were subsequently integrated into three mucoadhesive gel formulations, with the ultimate goal of determining the most efficacious system for the management of periodontitis. The formulations were assessed for enhanced mucoadhesion, sustained drug release, reduced crystallinity, and improved antibacterial and anti-inflammatory efficacy. By leveraging the synergistic properties of Melox as a selective COX-2 inhibitor and liquid lipid as an antibacterial and anti-inflammatory agent, this work seeks to advance localized drug delivery systems for prolonged retention and therapeutic effectiveness in periodontal pockets.

## 2. Materials and Methods

### 2.1. Materials

Compritol^®^888 ATO (S.L; glycerol dibehenate), clove oil (CO, *Eugenia* spp. (76.8% eugenol)), Miglyol^®^812N (Mig; triglyceride ester of saturated caprylic and capric fatty acids), and Kolliphor^®^ RH40 (PEG-40 castor oil, ethoxylated, Surfactant) were received from Gattefossé (Saint-Priest Cedex, France), Sigma-Aldrich (Steinheim, Germany), Sasol GmbH (Witten, Germany), and BASF SE Chemtrade GmbH (Ludwigshafen, Germany), respectively. Potassium phosphate (KH_2_PO_4_), Mucin (porcine gastric grade III), and Meloxicam (4-hydroxy-2-methyl-n-(5-methyl-2-thiazolyl)-2H-benzothiazine-3-carboxamide-1,1-dioxide) were supplied by Sigma Aldrich Co., Ltd. (Budapest, Hungary). Hypromellose (HPMC; Methocel F4M) was sourced from Colorcon (Dartford, UK). ZnHA (Mol. wt.: 498 kDa) and NaHA (Mol. wt.: 1400 kDa) were kindly acquired from Gedeon Richter Plc. (Budapest, Hungary). The lyophilized form (≥97% pure) of bovine serum albumin (BSA) was obtained from SERVA Electrophoresis GmbH (Heidelberg, Germany). We purchased 85% orthophosphoric acid and 100% ethanol from VWR International (Debrecen, Hungary). For experiments purified water was filtrated through a Millipore Milli-Q^®^ 140 Gradient system (Merck, Ltd., Budapest, Hungary). All the materials and chemicals were of analytical grade.

### 2.2. Methods

#### 2.2.1. Preliminary Study

A preliminary study was conducted to establish a foundational understanding of the development of the optimized NLC formulation. Guided by insights from the literature, several initial formulations were prepared by varying the lipid concentration up to 5% and adjusting the surfactant concentration in surfactant-to-lipid ratios of 0.5, 0.8, and 1. These formulations were evaluated for key physicochemical parameters, including particle size (Z-average), polydispersity index (PDI), and zeta potential (ZP), as mentioned in the Supplementary Materials (Table S1). The outcomes of this initial screening resulted in the selection of composition ranges for further systematic formulation development and investigation.

Formulation A was selected as the optimal formulation based on its superior physicochemical characteristics, including a more favorable ZP (−19.5 mV), a relatively low PDI (0.26), and an acceptable particle size (Z-average of 183.0 nm). Notably, these parameters were achieved using the lowest concentration of surfactant (2.5%) among the tested formulations. This optimized formulation was subsequently selected for drug loading in further studies.

#### 2.2.2. NLC Preparation

NLC–Melox was prepared by the ultrasonication method, where the lipid phase was prepared with solid and liquid lipids (S.L and CO, respectively), Melox, and surfactant. The lipid phase was heated at 85 °C (liquid lipid was added at the end just before mixing both phases). On the other hand, the heated aqueous and lipid phases were gradually mixed and the subsequent pre-emulsion was subjected to ultrasonication with Hielscher UP200S ultrasonic homogenizer (Hielscher Ultrasonics GmbH, Teltow, Germany) at 70% amplitude for 5 min continuously to obtain the nanosized dispersion. The obtained nanodispersions were cooled in ice and stored in air-tight containers.

**Figure 1:**
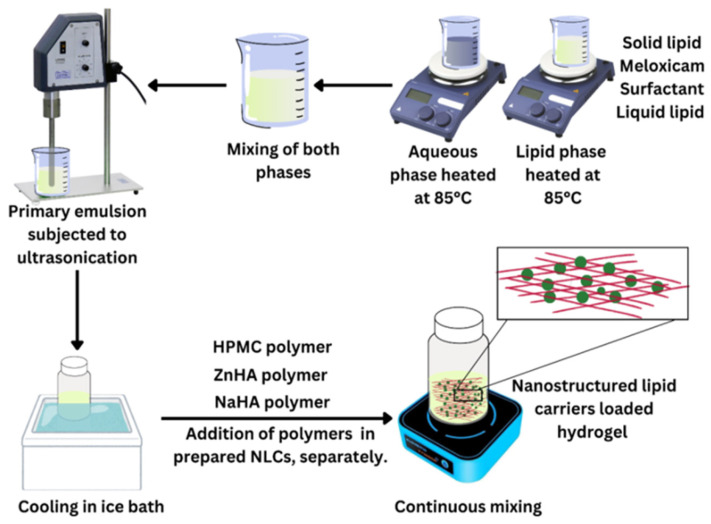
Preparation of NLC–Melox and NLC–Melox-loaded 3 different types of polymeric hydrogel systems.

Later, the NLC–Melox was continuously stirred separately with polymers to obtain 3 different types of homogenous nanodispersion-loaded polymeric hydrogels (Figure 1). The components of nanodispersions and all the polymeric gel systems are provided in Table 1.

Lyophilization was performed for thermal, crytallographic, and spectroscopic analysis using Scanvac CoolSafe 100-9 Pro equipment (LaboGene ApS, Lynge, Denmark). For the first drying cycle, small vials containing NLC–Melox and NLC-loaded polymeric gels were placed in a 0.015 mbar pressure chamber at −40 °C for 24 h, following the secondary drying at 25 °C for the next 12 h to obtain lyophilized powders. The whole process of temperature and pressure control was operated with the Scanlaf CTS16a02 program. Obtained lyophilized samples were stored in a refrigerator.

#### 2.2.3. Dynamic Light Scattering Measurements of NLC–Melox

Z-average, PDI, and ZP of the nanoparticles were determined at 633 nm laser wavelength with a Zetasizer Nano ZS instrument (Malvern Instruments, Worcestershire, UK). For the analysis, DTS 1070 folded capillary cells were used with diluted samples (10 × dilution with water), at 110 s equilibration time and 25 °C. All data points were presented as means ± SD, where n = 3.

#### 2.2.4. Morphological Analysis (Transmission Electron Microscopy (TEM))

All the prepared formulations (NLC–Melox, NLC-HPMC gel, NLC-ZnHA gel, NLC-NaHA gel) were microscopically inspected with a JEM-1400 Flash transmission electron microscope (JEOL, Tokyo, Japan) to identify their morphology and distribution with some modifications to the reported method [26]. For preparing the samples for the staining procedure, 10 µL of the samples (diluted 100-fold) were applied onto a formvar-coated 150-mesh copper grid (Electron Microscopy Sciences, Hatfield, PA, USA). Following a 1 min rest, excess liquid was carefully cleared with the filter paper. Afterward, the samples were contrasted with 10 µL of 2% uranyl acetate (Electron Microscopy Sciences Hatfield, PA, USA) in Millipore water for 2 min (2 times). After eliminating the surplus staining solution, the samples were allowed to dry overnight under a Petri dish before electron microscopic assessment. The negatively stained specimens were meticulously screened at magnifications spanning 5000× to 20,000× to precisely detect and map the presence of nanoparticles and subsequently recorded at 25,000–50,000× magnification (200 nm, 100 nm bar scale) with a 16 MP Matataki Flash camera (JEOL, Tokyo, Japan). TEM analysis was repeated after 3 months of storage to evaluate the long-term stability and morphological integrity of nanoparticles incorporated into different gelling systems.

#### 2.2.5. Entrapment Efficiency (EE%) and Drug Loading (DL%) of Melox

The unentrapped Melox from nanocarriers and nanocarrier-loaded gels was separated through centrifugation at 16,000 rpm for 30 min at 5 °C using Vivaspin 15R 5 kDa molecular weight cut-off (MWCO) Hydrosart™ tubes (Sartorius, Stonehouse, UK) with Hermle Z323K (HERMLE Labortechnik GmbH, Wehingen, Germany), a high-speed centrifuge machine with cooling system. The clear aqueous phase was collected to quantify the free amount of drug with HPLC (Section 2.2.11) and to calculate EE% by Equation (1). DL% was determined by Equation (2).

(1) $$EE\%=\frac{W_{loaded\;Melox}-W_{free\;Melox}}{W_{loaded\;Melox}}\times 100$$

(2) $$DL\%=\frac{W_{loaded\;Melox}-W_{free\;Melox}}{W_{nanoparticles}}\times 100$$

In the above equations, W is denoted for weight.

#### 2.2.6. Thermal Analysis (Differential Scanning Calorimetry)

The melting behavior of all the samples and raw materials was investigated by using a Differential Scanning Calorimetry (DSC) thermal analysis system (Mettler-Toledo 821e DSC, Mettler-Toledo GmbH, Greifensee, Switzerland), operated by STARe thermal analysis software version 16.30. Sample analysis was performed using a 40 μL holed aluminum pan, also serving as a reference pan. All Melox-containing samples (weighed between 2 and 5 mg) were treated from 10 °C to 350 °C at a rate of 10 °C/min under a nitrogen flow (80 mL/min) [27]. In the case of S.L, the heating range was selected from 0 °C to 100 °C at the rate of 5 °C/min and 40 mL/min of N_2_ flux. Data were normalized relative to the sample’s weight for analysis. Based on the DSC enthalpy measurements, the crystallinity index (CI%) of the nano compositions was determined using Equation (3).

(3) $$CI\%=\frac{Enthalpy\;of\;NLC\;or\;NLC-gel\left[\frac{J}{g}\right]}{Enthalpy\;of\;bulk\;material\left[\frac{J}{g}\right]\times Lipid\;phase\left(concentration\right)\left[\%\right]}\times 100$$

#### 2.2.7. Crystallographic Analysis (X-Ray Diffraction)

The drug’s transition from a crystalline to an amorphous or dissolved state within the formulation was evaluated with X-ray Diffraction (XRD). Analysis was performed on each sample (pure Melox, S.L, physical mixture melt, NLC–Melox, and NLC-loaded gels).

The crystalline structure of all the components was examined using XRD with a BRUKER D8 Advance diffractometer (Bruker AXS GmbH, Karlsruhe, Germany) equipped with Cu Kα radiation (λ = 1.5406 Å) and a VANTEC 1 detector. The analysis was performed at 40 kV and 40 mA. The scan covered an angular range of 3° to 40° 2θ, with a step time constant of 0.1 s. Data processing and evaluation were performed using EVA Software version 13.0.0.1 (EVA Software Solutions, A223, Mumbai, India). To interpret the data, the diffractograms were compared to assess changes in peak positions and intensities.

#### 2.2.8. Raman Spectroscopy

Thermo Fisher DXR Dispersive Raman Spectrometer (Thermo Fisher Scientific. Inc., Waltham, MA, USA) was used in order to explore potential interactions between the additives, Melox, NLC–Melox, and NLC–Melox-loaded polymeric materials. The instrument was integrated with a charged-coupled device (CCD) and a diode laser at a wavelength of 780 nm and a range of 3000–200 cm^−1^. Raman measurements were performed with a laser power of 24 mW, using a slit aperture of 25 µm, focusing on a 2–3 µm spot size with an estimated resolution of 2.4–4.4 cm^−1^. The spectra of the samples were recorded with an exposure time of 6 s and an exposure count of 24. Data acquisition and evaluation were carried out using the OMNIC™ 8.2 for Dispersive Raman software 8.3.104 package by Thermo Fisher Scientific Inc. (Waltham, MA, USA). Normalization of the Raman spectra was performed to correct the variations in signal intensity observed between the measured areas.

#### 2.2.9. Mucoadhesive Study

Mucoadhesion testing was conducted using a TA.XT plus Texture Analyzer (Stable Micro Systems Ltd., Surrey, UK) equipped with a 5 kg load cell. A 50 µL aliquot of mucin dispersion (8% *w*/*w*) was applied to a wetted filter paper (Whatman^®^ qualitative filter paper, Sigma Aldrich Co. Ltd., Budapest, Hungary) and positioned in the mucoadhesive test rig. Both the mucosal surface and the test rig were maintained at 35 °C throughout the measurements. In total, 20 µL of the sample was positioned on a cylindrical probe of a 10 mm diameter. The probe was lowered at a rate of 0.3 mm/s to bring the mucin layer and sample into contact. Following the contact, a preload of 2500 mN was applied for 3 min. Subsequently, the probe was raised at a rate of 2.5 mm/min to detach it from the mucosal surface, thereby breaking the adhesive bond [2]. This procedure was repeated five times to evaluate the adhesive force and work, which are indicative of the mucoadhesive properties of the hydrogels.

#### 2.2.10. Rheological Analysis

Rheological measurements (loss and storage moduli) were conducted using a Physica MCR 302 Modular Compact Rheometer (Anton Paar, Graz, Austria) with a cone and plate measuring system (CP25-1, diameter: 25 mm, cone angle: 1°, gap: 0.05 mm). An amplitude sweep test was conducted to find the linear viscoelastic range (LVER) of polymeric samples. Frequency sweep tests were conducted over an angular frequency range of 0.1–100 rad/s within the LVER. For viscosity measurements, flow property analysis was initiated, where the rheometer continuously recorded the data, providing viscosity values as a function of the applied shear rate at 25 °C and 300 s of shearing time, ranging from 0.1 to 100 s⁻^1^ (up curve) and 100 to 0.1 s⁻^1^ (down curve). These findings were analyzed by graphing shear rate versus viscosity at several points. Measurements were performed in triplicate.

#### 2.2.11. In Vitro Release Study

The drug release behavior of all the samples and suspensions was evaluated by using the dialysis bag method. Cellulose dialysis membrane tubes (Zellutrans/Roth, Carl Roth GmbH+ Co., Germany) with dimensions of 10 mm width, 6.37 mm diameter, and a molecular weight cutoff (MWCO) of 12,000–14,000 Da were used. Each tube was filled with 0.7 mL of the sample and sealed using Spectra/Por^®^ Closures (Spectrum Laboratories, Inc., Rancho Dominguez, CA, USA). The filled tubes were weighed to ensure accurate weight-based calculations and then immersed in 10 mL of acceptor phase prepared with ethanol and PBS (pH = 7.4) in a 3:7 ratio. To ensure the sink conditions throughout the drug release study, the release medium was prepared to dissolve at least six times the amount of Melox loaded into the tubes. At specified intervals (0.5, 1, 2, 4, 6, 12, 18, and 24 h), 0.3 mL of the acceptor phase was withdrawn and replaced with a fresh medium to maintain the release environment. Each formulation underwent three parallel dissolution studies, and drug release profiles of the NLC–Melox, NLC-loaded polymeric gels, and Melox suspension were compared.

The samples collected during the study were analyzed using high-performance liquid chromatography (HPLC) with a Shimadzu Nexera X2 UHPLC system (Kyoto, Japan), a method applied with some modifications [28]. The mobile phase consisted of (A) 0.065 M KH_2_PO_4_ solution (pH 2.8) adjusted with 85% orthophosphoric acid and (B) 100% methanol. The selected stationary phase was a C18 reverse-phase column, Phenomenex Kinetex C18 column (2.6 μm, 150 mm × 2.1 mm, 100 Å by Phenomenex, Torrance, CA, USA) with an injection volume of 5 μL and an oven temperature of 40 °C, while the flow rate was set to 1 mL/min. Gradient elution was performed as follows: initially, the eluent was 70% of phase A, which was gradually reduced to 30% over the programmed 6 min. The retention time for the peak was observed as 3.6 min at a wavelength of 355 nm. The gathered data was analyzed to ascertain the release pattern.

The detection limit (DL) and quantitation limit (QL) were calculated by using the standard deviation (σ) of response and the slope (S) of the calibration curve. The regression coefficient (R^2^) of the calibration curve was 0.998, and the calibration was performed from a 100 μg/mL Melox standard stock solution prepared in methanol. DL was calculated using the formula DL=3.3 σ∕S, and QL was calculated as QL=10 σ∕S. The calculated values were 2.86 μg/mL and 8.67 μg/mL, respectively.

#### 2.2.12. Assessment of In Vitro Anti-Inflammatory Activity

The anti-inflammatory potential of the Melox and CO-loaded NLCs was evaluated based on their ability to inhibit heat-induced denaturation of BSA. A method mentioned in the literature was modified to conduct this anti-inflammatory assay [29]. For this experiment, 2-fold dilutions of the formulation (with a Melox concentration of 250–7.8 µg/mL and a CO concentration of 20,000–625 µg/mL) were prepared in distilled water. NLC–Melox, NLC- C (without Melox), and NLC-M (with Melox and without CO, where Mig was used as a liquid lipid) were prepared for the investigation. Unencapsulated Melox (aqueous suspension) and CO (oil in water emulsion using 1% polysorbate 20) with the same concentrations as in the final formulations were also investigated in this study to evaluate their inherent pharmacological activity. A 1% BSA solution (500 µL), prepared in phosphate-buffer saline (PBS) at pH 6.4 and adjusted with orthophosphoric acid, was combined with an equal volume of each sample. These combined mixtures were thoroughly mixed and incubated at 37 °C for 30 min initially, which was then followed by a second incubation at 70 °C for an additional 50 min in an AgroLab WB22 (Gongxiang, Kunshan, China) water bath. After the incubation, the samples were cooled down to room temperature, and their turbidity was measured spectrophotometrically at 660 nm using a Thermo Scientific Evolution 201 Ultraviolet-Visible Spectrophotometer (Thermo Fisher Scientific, Waltham, MA, USA). The instrument was operated using the Thermo Insight v1.4.40 software package. A BSA solution without formulations served as the control and the percentage inhibition of the BSA denaturation assay was calculated using Equation (4) [30].

(4) $$Inhibition\%=\frac{\left({Abs}_{c}-{Abs}_{s}\right)}{{Abs}_{c}}\times 100$$

where Abs_c_ represents the absorbance of the control and Abs_s_ represents the absorbance of the test samples. This formula quantifies the extent to which the Melox-loaded formulations prevented protein denaturation under heat-induced conditions.

#### 2.2.13. In Vitro Antibacterial Study (Bacterial Cell Culture)

The antibacterial activity was determined by using *Aggregatibacter actinomycetemcomitans* (*A. actinomycetemcomitans*) DSM 11,122 and *Streptococcus mutans* (*S. mutans*) ATCC 25,175 as reference strains. Both strains were cultured on Columbia agar supplemented with 5% sheep blood (Biomerieux, Marcy-l’Etoile, France) and incubated at 35 °C in a 5% CO_2_ atmosphere for 24 h. Bacterial suspensions were prepared by directly inoculating the colonies into sterile 0.85% NaCl solution, with the turbidity adjusted to 0.5 McFarland (approximately 1 × 10^8^ CFU/mL).

##### Agar Well Diffusion Method

Specimens were uniformly streaked onto Columbia agar plates enriched with 5% sheep blood. In total, 200 µL of test samples including NLC–Melox, NLC loaded gels, blank gels, NLC-C, and CO emulsion (prepared with 1% Polysorbate 20 to ensure uniformity), filled in screw caps (designed for HPLC: 12 mm outer diameter), were positioned on the agar surface in triplicate. The plates were incubated at 35 °C under 5% CO_2_, and the size of the inhibition zone (mm) was recorded at 24 and 48 h. Melox aqueous suspension and NLC-M served as negative controls, as Melox has no documented antibacterial activity.

##### Minimum Inhibitory Concentration (MIC)

A microdilution broth assay was performed in 96-well plates to ascertain the MIC of the free and encapsulated therapeutic agent, following the EUCAST protocol [31]. Serial 1:1000 dilutions of each formulation were prepared using distilled water as a diluent. Following this, each well received 100 µL of a bacterial suspension (approximately 1 × 10^5^ CFU/mL) in brain heart infusion (BHI) broth (Oxoid, Basingstoke, UK).

For the CO-containing formulations, two-fold serial dilutions resulted in final concentrations ranging from 20,000–78.12 µg/mL. Distilled water served as the negative control. Furthermore, each dilution was combined with BHI broth to accommodate the intrinsic turbidity of the nanoparticles. Positive controls consisted of BHI broth with a bacterial suspension and chlorhexidine, the latter tested in concentrations from 0.008 to 0.25 µg/mL using two-fold serial dilutions. All the samples were incubated at 37 °C in a 5% CO_2_ environment for 24 h. After incubation, the optical density was assessed at 620 nm. Each experiment was replicated three times.

##### Minimum Bactericidal Concentration (MBC)

To determine the lowest concentration of the samples capable of killing both bacterial strains, 10 μL of broth dilutions from the MIC microplate wells were cultured on Columbia agar supplemented with 5% sheep blood (Biomerieux, Marcy-l’Étoile, France) and incubated at 35 °C under 5% CO_2_ for 24 h [32].

#### 2.2.14. Stability Study

In order to ensure the stability of formulations, NLC–Melox was kept in airtight glass vials in a controlled temperature (4 ± 2 °C) environment. The formulation was evaluated after 3 months for critical parameters, including Z-average, PDI, and ZP by DLS method as detailed in Section 2.2.3. TEM analysis was conducted after 3 months of storage under the same conditions to evaluate the long-term stability of nanoparticles incorporated within the gelling system, in terms of morphological integrity, particle dispersion, and potential aggregation (Section 2.2.4). These measurements provide critical insights into structural stability over time.

#### 2.2.15. Statistical Analysis

The data in triplicate are presented as means ± standard deviation (SD), wherever applicable. Graph generation and statistical analyses were performed using GraphPad Prism 8.0.2 for Windows (GraphPad Software, Inc., La Jolla, CA, USA) and OriginPro^®^ 8.6 (OriginLab Corporation, Northampton, MA, USA). Differences between groups were assessed by student t-test and among groups using one-way variance analysis (ANOVA) followed by Tukey’s post hoc test for multiple comparisons.

## 3. Results and Discussion

### 3.1. Dynamic Light Scattering

DLS is a reliable in situ measurement technique that provides statistical data on the Z-average (as small as a few nanometers), PDI, and ZP of nanocarriers [33]. The development of Melox-loaded polymeric hydrogels for periodontal therapy represents a significant advancement in addressing the dual challenges of chronic inflammation and tissue destruction in periodontitis. The findings of this study align with and expand upon the existing literature, offering insights into the design of effective localized drug delivery systems.

To ensure an ideal formulation behavior in terms of stability, targeted delivery, distribution, and safety, nanoparticle size and dispersion are essential characteristics in pharmaceutical applications [34]. Z-average plays a crucial role in enhancing drug solubility, improving bioavailability, and formulation stability during both processing and storage [35].

NLC–Melox nanoparticles exhibited a Z-average of 183 ± 3.0 nm, which is a critical factor for predicting drug release kinetics as nanoparticles affect the release of drugs. According to the literature, the diminutive size of nanoparticles confers a significantly elevated surface-area-to-volume ratio, a characteristic that profoundly influences drug loading dynamics and release. In smaller nanoparticles, a substantial proportion of the encapsulated drug is localized at or proximate to the particle’s surface, susceptible to premature drug release [36]. Thus, only the optimized size of nanoparticles can better serve as drug depots for prolonged and controlled drug release [37].

PDI is a key metric in the pharmaceutical field, essential for evaluating the uniformity and stability of nanoparticle size distribution within the system. NLC–Melox exhibited a non-agglomerated system with a PDI of 0.26 ± 0.01. A PDI value below or equal to 0.3 is generally considered to indicate the presence of homogeneous particles [38].

A ZP of −24.7 ± 1.5 mV indicated colloidal stability, likely due to steric stabilization by surfactants, which prevented aggregation. The Derjaguin Landau Verwey Overbeek (DLVO) electrostatic theory explains the stability of emulsions and colloids by balancing between attractive forces (van der Waals) and repulsive forces. Due to lower ZP, the attractive forces become dominant, leading to the aggregation of particles, while higher values of ZP lead to higher electrostatic repulsion that promotes physical stability by preventing particle aggregation [39]. After getting satisfactory results of NLC–Melox with the dynamic light scattering method, nanoparticles were loaded into polymeric solutions and subjected to microscopy to check the particle size, shape integrity, and particle distribution.

### 3.2. Transmission Electron Microscopy

NLCs loaded in hydrogels were analyzed by TEM, which is a powerful imaging technique used to visualize the internal structure of materials at the nanoscale. The surface morphology of lipid-based particles was assessed in roughly spherical and ellipsoidal shape nanoparticles [40].

**Figure 2:**
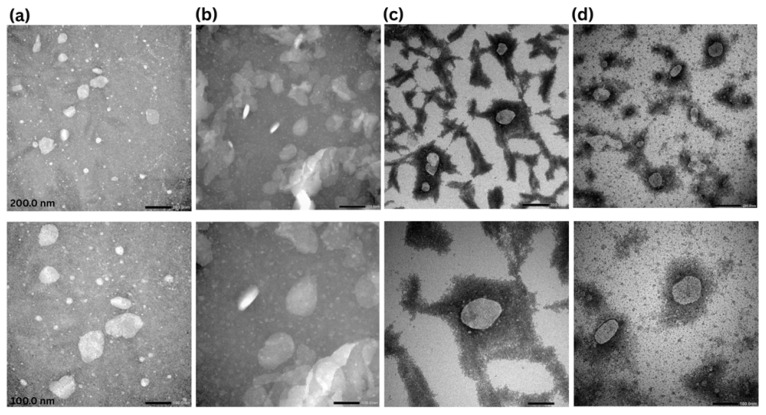
TEM images of Melox-loaded nanoparticles and gel formulations at 25,000 and 50,000× magnifications with scale bars 200 nm (1st row) and 100 nm (2nd row). (**a**) NLC–Melox, (**b**) NLC-HPMC gel, (**c**) NLC-NaHA gel, and (**d**) NLC-ZnHA gel.

The diameter distribution of nanoparticles ranged up to 157 nm, which not only corresponds to the nanosizing claim but is also nearly the same as DLS measurements. The difference in particle size is justified as TEM measures the diameter in the dried state, while DLS calculates the Z-average in the hydrated state (solvation layer included) [34]. Upon the addition of NLC–Melox to gelling systems, the particle size ranged from 115 nm to 157 nm. Furthermore, these images revealed no evidence of nanoparticle aggregation, showcasing a relatively uniform size distribution, which underscores the stability of the formulations, as shown in Figure 2.

Overall, these formulations demonstrated consistent homogeneity without detectable aggregates within the nanometric particle size range. Notably, the nanoparticles were observed to be distributed across the polymeric bed. Furthermore, the consistent retention of NLC–Melox particle size and shape following the incorporation into hydrogels (HPMC, ZnHA, NaHA) underscores the structural integrity and robustness of the formulations. This stability is a critical attribute, ensuring their potential for successful clinical translation.

**Figure 3:**
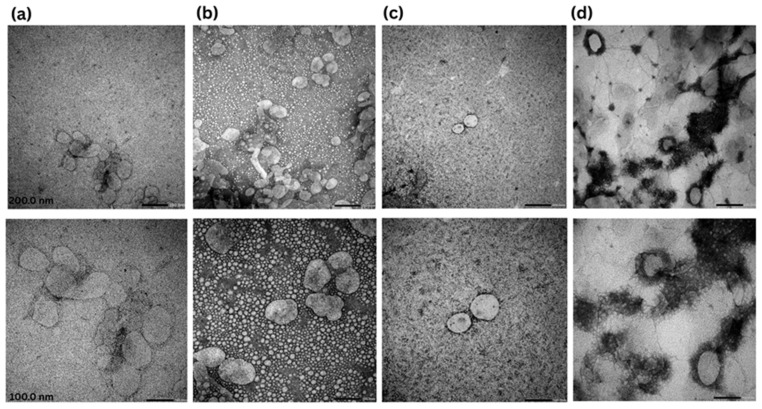
Stability study after 3 months, TEM images of Melox-loaded nanoparticles and gel formulations at 25,000 and 50,000× magnifications with scale bars 200 nm (1st row) and 100 nm (2nd row). (**a**) NLC–Melox, (**b**) NLC-HPMC gel, (**c**) NLC-NaHA gel, and (**d**) NLC-ZnHA gel.

After 3 months of storage, TEM images (Figure 3) showed that NLC–Melox maintained its original particle morphology and dimensional characteristics, yet exhibited formation of loose aggregates.

In contrast, nanoparticles incorporated into the HPMC gel matrix displayed a homogeneous distribution with reduced aggregation. Similarly, NLC–Melox in NaHA showed well-dispersed morphology by retaining its original shape without significant deformation. NLC-ZnHA gel showed intact structure and homogeneous dispersion, further reinforcing the protective role of the gel network. Overall, these results confirm that nanoparticles, before and after incorporation into gelling systems (HPMC, NaHA, and ZnHA), maintained their structural integrity and dispersion stability over time, with no significant aggregation or shape alterations.

### 3.3. Drug Loading and Entrapment Efficiency

The EE% and DL% values for NLC–Melox and NLC formulations loaded into different gel matrices were evaluated. The standard NLC–Melox formulation demonstrated an entrapment efficiency of 91.6% and a drug loading of 4.57%. The enhanced EE observed in the NLC–Melox can be attributed to the combination of solid and liquid lipids, which results in a less ordered matrix. This imperfect structure creates additional space, facilitating greater drug incorporation. Additionally, the drug demonstrated improved solubility in liquid lipids compared with solid lipids, which further enhanced EE% [41].

Upon incorporation into gel-based systems, the EE% and DL% improved. All three polymers exhibited commendable DL capacities, with ZnHA and NaHA slightly outperforming HPMC. The NLC–Melox-HPMC gel exhibited an EE% of 97.9% and a DL% of 4.89%. Similarly, NLC–Melox-ZnHA gel and NLC–Melox-NaHA gel formulations achieved exceptionally high EE% (99.9% and 99.8%, respectively) and drug loading capacities (4.98% and 4.99%, respectively). These results indicate that after integrating NLC–Melox (without purification) into hydrogels, the drug (free form) remained intact in the polymer system, potentially contributing to the initial release followed by controlled release from NLCs. This optimization may contribute to better therapeutic outcomes in drug delivery applications.

### 3.4. Differential Scanning Calorimetry

**Figure 4:**
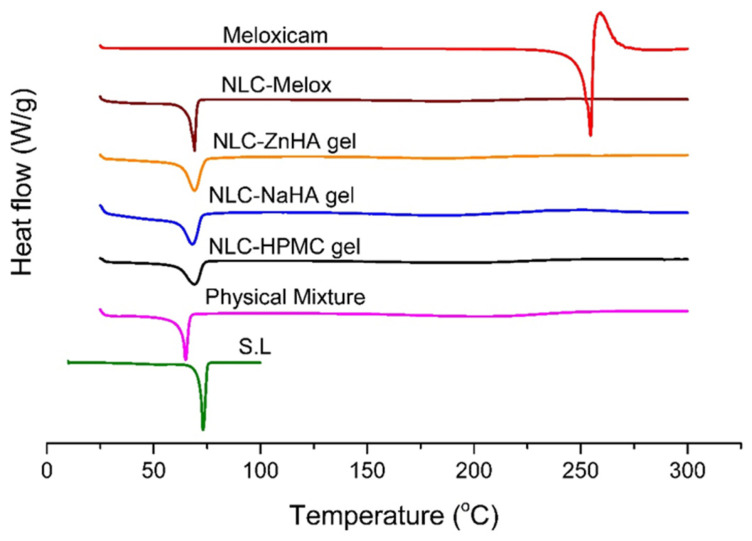
DSC thermograms of the pure drug, crystalline lipid, physical melt, Melox-loaded NLC, and polymeric gel systems to present the amorphous form of the lipid and the complete miscibility of Melox in the matrix.

DSC is a thermodynamic technique that analyzes the thermal behavior of materials by measuring heat flow during transitions relative to temperature or time. The thermograms of solid lipid, pure drug, physical mixture, NLC–Melox, and NLC–Melox-loaded gels are presented in Figure 4. The melting point for pure Melox was shown to be 253.5 °C, which was closer to the stated value in literature displaying Melox crystalline characteristics [42]. As Melox was solubilized in the excipients, its characteristic peak in the physical mixture and formulations disappeared.

The DSC of Melox revealed a distinct endothermic peak at 253.5 °C, which is the melting point of Melox in its crystalline form. After melting, the scan displayed an exothermic peak (260 °C) that is an indication of Melox instability attributed to oxidation, deterioration, or degradation as mentioned by researchers, who linked this exothermic peak to Melox degradation [27]. On the other hand, a distinct endothermic melting peak of S.L was visible at 73.05 °C. The conversion of bulk material into nanoparticles alters the melting behavior of the lipid, often resulting in the emergence of lower-melting polymorphic forms. A decrease in the melting temperature and an increase in the cohesive force could be a possible reason for nano range particle size [43]. That is why S.L had less pronounced endothermic melting peaks in different formulation scans, while Melox did not exhibit any endothermic peak. DSC parameters are detailed in Table 2, where the percent crystallinity index of formulations ranged from 16.3% to 22.4%.

The decrease in crystallinity can be attributed to the partial formation of lower-energy lipid polymorphs. Additionally, surfactants incorporated into the melted lipid phase during production can disrupt crystallization, leading to a reduction in melting enthalpy [44].

The widening of the heating peak and the lowering of the melting point may be because of an increase in lattice imperfections [45]. DSC analysis revealed that the enhanced dissolution of the drug could be attributed to its transformation from a crystalline to an amorphous state within the lipid matrix, as demonstrated by the DSC results. The absence of additional peaks in DSC further validated the safe drug–excipient interactions, thus ensuring the stability of the system—a finding consistent with Rai et al., who reported that no interactions or extra peaks appeared in the NSAID-loaded polymeric system. This thermal resilience is pivotal for long-term storage and practical application in varying environmental conditions [46].

The absence of the Melox peak in the physical melt was due to its solubilization within the excipients. Upon encapsulation into the nanoparticles, the drug was entirely transformed into an amorphous state. DSC thermograms confirmed the amorphous or dissolved state of Melox in nanoforms and hydrogels, as evidenced by the absence of crystalline melting endotherms. Amorphization is known for enhancing drug solubility by circumventing the energy-intensive dissolution of crystalline lattices [47].

### 3.5. X-Ray Diffraction

**Figure 5:**
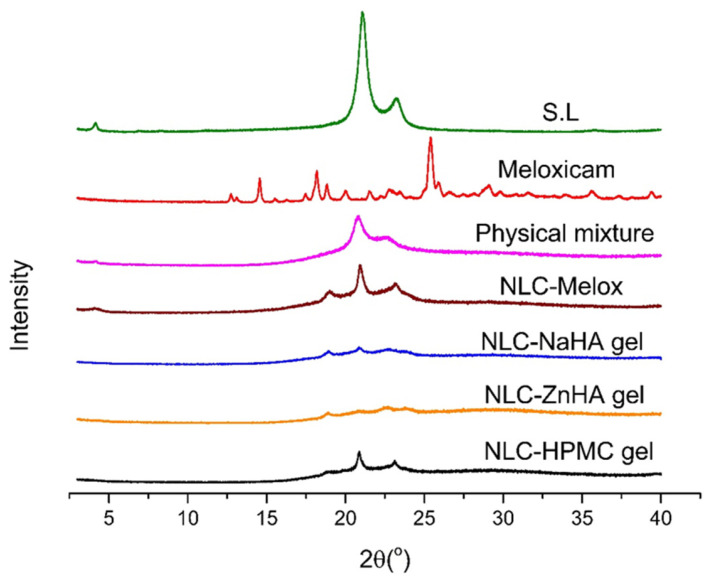
XRD spectra of the solid lipid, the physical mixture (melt), Melox, NLC–Melox, and NLC–Melox-loaded polymeric gels, emphasizing the excipient–drug compatibility and complete solubilization of Melox after its incorporation into the nanodelivery system.

XRD is an analytical technique used to study the structure of crystalline materials by measuring the diffraction patterns produced by X-ray interaction with the lattice structure of the material. To assess crystallinity, the diffractograms of the lipid, pure Melox, the physical mixture, and the formulations were obtained and compared. The pure drug exhibited sharp, prominent peaks at 2θ values of 14.58°, 18.19°, 25.42°, 29.09°, and 35.53°, confirming its crystalline nature. Solid lipid has its characteristic peaks at 4.00°, 21.89°, and 23.22°, as shown in Figure 5.

In the physical mixture melt, characteristic peaks of the S.L were observed at the same 2θ values as in the XRD spectrum of S.L, with no significant high-intensity peaks, showcasing the compatibility of the carrier materials. Triglycerides can form three polymorphic structures, including the unstable α form, the metastable β′ form, and the most stable β form [48]. An intermediate structure, known as the βi form, exists between the β′ and β modifications. For the formulations, the 2θ values of lipids were recorded at approximately 18.90°, 20.90°, and 22.70°, with a significant reduction in crystallinity. The appearance of a new peak at 18.90° may serve as evidence for the partial formation of the βi polymorphic form, which observation is consistent with findings reported in prior research [49]. Furthermore, the alteration in peak intensities of S.L was likely attributed to the transformation of crystalline Melox into an amorphous state following its encapsulation within the lipid matrix.

Thus, XRD patterns corroborated the amorphous nature of Melox with the complete absence of the Melox characteristic peaks; this finding could be an indication of increased solubility of the drug within the matrix, which aligns with a previous study, where amorphous nanoparticles improved the solubilization and bioavailability of Melox [50]. These findings support our DSC results, where the amorphous/dissolved state of meloxicam was suspected.

### 3.6. Raman Spectroscopy

Raman spectroscopy relies on inelastic light scattering, where incident photons interact with molecules, causing a shift in photon energy equivalent to the vibrational energy levels of the molecules [51]. Spectral analysis of all raw materials was performed, where the most prominent Raman peaks of S.L appeared at 1061 cm⁻^1^ (asymmetric C-C stretching), 1128 cm⁻^1^ (symmetric C-C stretching), 1293 cm⁻^1^ (CH_2_ twisting), and 1428 cm⁻^1^ (CH_2_ scissoring) [52,53].

**Figure 6:**
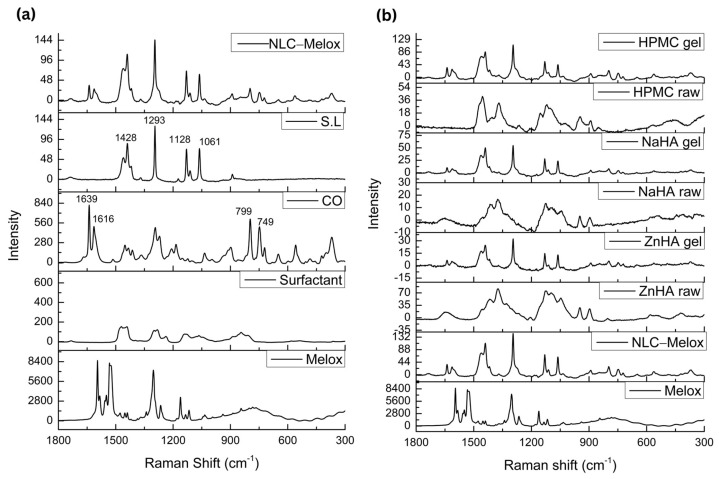
Obtained spectra of Raman scattering. (**a**) Raman spectra of excipients (CO, surfactant, S.L, Melox), and NLC–Melox. CO: The vibration of C=C bond stretching (1639 cm^−1^), ring quadrant stretching (1616 cm^−1^), aromatic CH wagging (799 cm^−1^), and ring quadrant in-plane bending mode (749 cm^−1^) are marked; S.L: C-C stretching (1061 cm^−1^, 1128 cm^−1^), CH twisting and scissoring (1293 cm^−1^, 1428 cm^−1^); (**b**) Raman spectra of NLC–Melox-loaded gels (HPMC, NaHA, and ZnHA) compared with raw polymers, Melox, and NLC–Melox, where no extra peaks or interactions were found.

Eugenol, the main component in CO, has a double bond that is expected to have a corresponding peak around 1680–1600 cm⁻^1^, and a strong Raman signal has appeared in the CO spectrum around 1639 cm⁻^1^ (C=C stretching). The much weaker signal at 1670 cm⁻^1^ can be linked to the C=C stretching of β-caryophyllene. A weak band at 1369 cm⁻^1^ in the spectrum was assigned as the O-CH_3_ wagging mode, which typically falls in the range of 1390–1340 cm⁻^1^. Additionally, a strong band at 1293 cm⁻^1^ can be linked to the =CH rocking mode, which is expected in the range of 1309–1288 cm⁻^1^ [54,55]. Eugenol contains an ether functional group, which typically produces specific Raman bands of Ar-O (Ar = benzene) stretching vibration between 1310 and 1210 cm⁻^1^ and the C-O stretching vibration between 1050 and 1010 cm⁻^1^. Furthermore, the bands at 1273 cm⁻^1^ and 1036 cm⁻^1^ can be attributed to the Ar-O and C-O stretching modes of eugenol [56], while a wagging vibration of the monoalkyl ethylene (R-CH=CH_2_) can be observed around 910 cm⁻^1^ in the Raman spectrum [57], as shown in Figure 6a,b.

Raw Melox exhibited characteristic vibrational modes in its Raman spectrum, including C=O stretching, C=C stretching in the range of 1600–1400 cm⁻^1^, C-C stretching between 1300 and 1200 cm⁻^1^, and C-S stretching between 1200 and 800 cm⁻^1^ that provide insights into its molecular structure and functional groups [58]. The absorption peaks predominantly observed in the range of 1600–1000 cm⁻^1^ with strong intensity were primarily attributed to vibrations of the aromatic ring structure [59].

In the case of HA-based raw polymers, the Raman bands in the range of 1200–1000 cm⁻^1^ primarily arose from C-O and C-C stretching vibrations. The peaks at 1092 cm⁻^1^ and 900 cm⁻^1^ can be attributed to the antisymmetric and symmetric C-O-C vibrations of β-glycoside linkages, respectively [60]. The HPMC polymer presented peaks at 1110 cm⁻^1^ (symmetric C-O-C), 1360 cm⁻^1^ (COH bending), and 1450 cm⁻^1^ (CH_2_ scissor functional groups) [61]. Overall, no new peaks or shifts in signal positions were observed in all these spectra, when comparing the formulations to excipients and raw materials.

Raman spectroscopy was employed to assess the molecular interactions and structural integrity of Melox within the formulation. Due to the low concentration of Melox in the primary formulation, the characteristic peaks of Melox were not distinctly visible, most likely due to their overlap with the spectral signals of the excipients and the low intensity of the drug’s Raman scattering. The overlapping or absence of Melox peaks in the primary formulation can also be attributed to the low drug-to-excipient ratio. However, to confirm the presence of Melox and evaluate potential interactions, additional formulations were prepared with an increased concentration of Melox. In these samples, the characteristic peaks of Melox were clearly observed, indicating that the drug retained its molecular structure without significant interactions with the excipients. The absence of new peaks or shifts in the Raman spectra suggested no chemical interaction between Melox and the additives. The Raman spectra of the samples with the added amount of Melox are provided as Supplementary Material (Figure S1) for further reference. These findings confirm that Melox remained chemically unaltered in the formulation, with its spectral features becoming evident at higher concentrations.

### 3.7. Mucoadhesive Study

Mucoadhesive force is a crucial characteristic to consider since it hinders the formulation’s quick escape from the cavity, ultimately affecting the bioavailability of the system. The degree of mucoadhesion bonding is influenced by the presence of functional groups, molecular weight, crosslinking, and the swelling behavior of polymers [62].

Hydrophilic functional groups (including hydroxyl and carboxyl groups) permit maximum exposure of possible anchor sites via hydrogen bonding with the substrate and swelling in an aqueous environment. Furthermore, the maximal chain distance found in swollen polymers results in enhanced chain flexibility and effective substrate penetration [63].

The augmentation of the mucoadhesive forces in the gel may be explained by the existence of hydrogen bond formation brought on by the quick absorption of fluid from the mucus layer (simulated mucosal membrane). As a result, the polymer chains can bind to the mucin chains by entering the mucin network. A series of phenomena occur for bioadhesion to take place. A bioadhesive polymer and a membrane come into close contact in the first step, either through bioadhesive swelling or wetting. Successively, after establishing the contact, the bioadhesive polymer penetrates the tissue surface’s crevices, or its chains entwine with the mucus [64].

**Figure 7:**
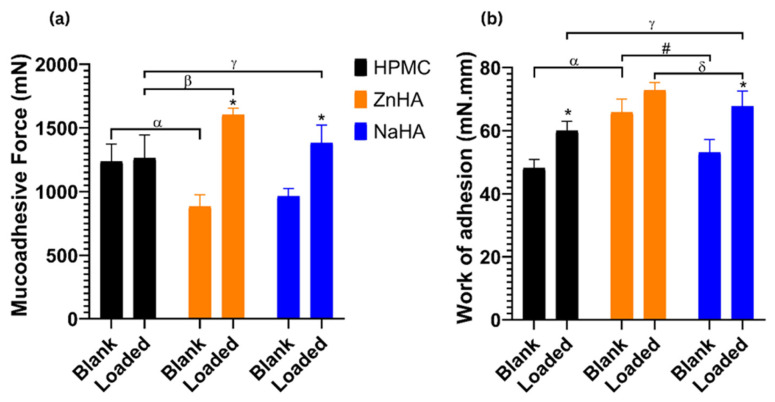
Comparison of (**a**) mucoadhesive force of NLC-loaded gels and blank gels, (**b**) work of adhesion of NLC-loaded gels and blank gels presented as means ± SD. Statistical significance between loaded and blank formulations of the same formulation was carried out by student-t test, * shows a significant difference between blank and loaded gels of the respective polymer (*p* < 0.05). Statistical significance among formulations of different polymers was carried out by one-way ANOVA followed by Tukey’s multiple comparison test; α shows a significant difference between blank ZnHA gel and blank HPMC gel; # shows a significant difference between blank ZnHA gel and blank NaHA gel; β shows a significant difference between loaded ZnHA gel and loaded HPMC gel; γ shows a significant difference between loaded NaHA gel and loaded HPMC gel; and δ shows a significant difference between loaded ZnHA gel and loaded NaHA gel (*p* ≤ 0.05).

A comparative analysis between blank gels and NLC–Melox-loaded gels revealed that the adhesion force was significantly lower in blank polymeric solutions compared with those containing the nanoformulation. The incorporation of NLC into the system enhanced the bioadhesive properties of the gelling system. In the case of comparison between three types of NLC-loaded gels, ZnHA achieved the highest mucoadhesive force (1604 mN), outperforming NaHA (1381 mN) and substantially exceeding HPMC (1236 mN) Figure 7a.

In alignment with our findings, blank gels demonstrated a consistent trend in mucoadhesive force, exhibiting significantly lower adhesive work than nanoparticle-loaded gels. The adhesive work values of blank gels, NLC-HPMC gel, NLC-NaHA gel, and NLC-ZnHA gel are displayed in Figure 7b, where NLC-ZnHA gel showed a higher level of work of adhesion (72.8 mN.mm) compared to NLC-NaHA gel (67.6 mN.mm) and NLC-HPMC gel (60 mN.mm).

ZnHA-based gels demonstrated superior mucoadhesive properties that can be attributed to zinc ions forming coordination bonds with mucosal glycoproteins. According to scientists, mucins have an average molar zinc binding capacity of over 200, suggesting that a single mucin molecule has many zinc-binding sites, potentially offering a wide range of distinct binding affinities [65]. Hence, combined strength and adhesion metrics make NLC-ZnHA gel more robust for bioadhesive applications.

### 3.8. Rheological Analysis

The rheological behavior was assessed by analyzing the frequency-dependent changes in the elastic storage modulus (G′) and the viscous loss modulus (G″) to determine the solid-like or fluid-like nature of gels [66]. The formulations exhibited viscoelastic characteristics. In contrast to NLC-loaded polymeric gels, blank gels behaved predominantly as a viscous fluid, with G″ exceeding G′ at some points. G″ was consistently lower than G′, indicating that overall solid-like behavior predominated in all 3 loaded gels examined.

**Figure 8:**
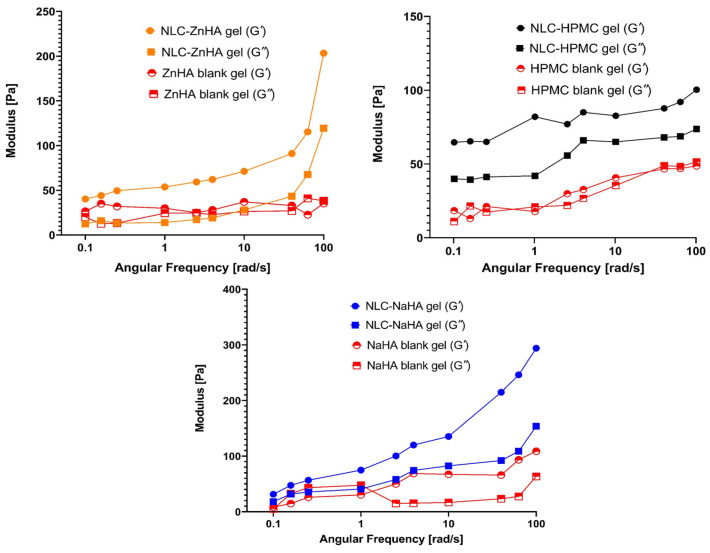
Frequency sweep curves showing storage (G’) and loss modulus (G”) of blank and NLC–Melox-loaded gels in relation to angular frequency. All the data points are presented as mean (n = 3).

This study offered a detailed comparative analysis of the rheological properties of ZnHA, NaHA, and HPMC polymeric gels with and without nanoparticles. In the case of all three blank gels, G′ and G″ were lower than those of the NLC–Melox-loaded gels (Figure 8), indicating that the integration of NLCs within the polymeric matrix effectively improved the rheological behavior of the system. Furthermore, findings revealed that NaHA gel exhibited the highest storage modulus (G′ = 293 Pa) and loss modulus at higher frequencies, indicating superior structural integrity and elasticity.

The HPMC and ZnHA blank gels showed viscoelastic liquid (G′ ≈ G″) behavior, while the NaHA blank gel represented an elastic behavior at high frequency and a liquid behavior at low frequency with a cross-over point at approximately 1 rad/s angular frequency. This rheological behavior is generally known for hyaluronates and is basically related to the relaxation of the chains. This property disappeared with the addition of nanolipids, the elastic properties dominated in the entire frequency range. In the case of the Zn salt, this crossover point was not observed, and G’ and G” were almost equal, behaving as a viscoelastic liquid, which also became an elastic gel with the addition of the NLC.

The injectability of formulations can be characterized by viscosity curves. A shear-thinning behavior observed in hyaluronates, where viscosity decreases with increasing shear rate, is characteristic of polymeric systems with strong intermolecular interactions [67]. HPMC, while also being shear-thinning, showed a less dramatic increase in viscosity at lower shear rates, indicating a less dense or less entangled network. These differences in viscosity and shear-thinning behavior can be attributed to the molecular weight, crosslinking density, and hydrophilicity of each polymer. The high viscosity and strong shear-thinning properties of NaHA make it suitable for applications requiring structural integrity under low stress but ease of flow under high stress, such as injectable formulations [68].

**Figure 9:**
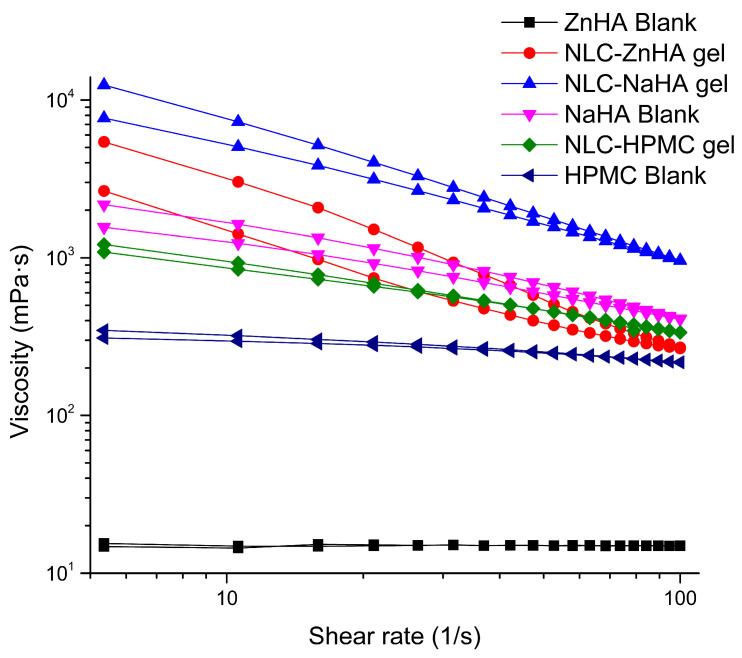
Viscosity curves of polymeric gelling system with and without NLC–Melox (Mean, n = 3).

The NLC-ZnHA gel, with intermediate viscosity, may offer a balance between mechanical strength and flowability, while the lower viscosity of the NLC-HPMC gel suggested that it is better suited for applications requiring less resistance to flow. In the case of each polymeric solution, the viscosity of the blank gels was notably lower than that of the NLC-loaded gels, which further confirmed that the presence of NLCs contributed to improved structural integrity and flow behavior in the polymeric system as seen in Figure 9.

### 3.9. In Vitro Release Study

**Figure 10:**
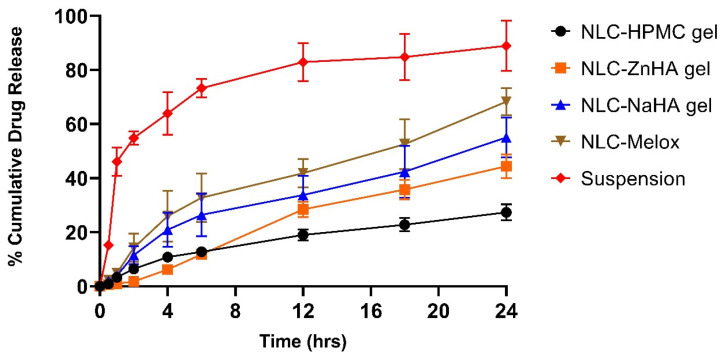
In vitro release study of formulations and Melox suspension showing the % cumulative release of Melox, calculated as Mean ± S.D (n = 3).

The release profiles of NLC–Melox, Melox suspension, and Melox-loaded gels (HPMC, ZnHA, and NaHA) were evaluated over 24 h. From the beginning, the Melox suspension exhibited a faster release (15.24%) compared with NLC–Melox (2.35%) due to the diffusion of the acceptor phase into the donor phase and the dissolution of the Melox particles. This trend continued until 24 h, where 88.91% of the drug was released from the suspension, while NLC–Melox showed a 68.27% cumulative release during the same period. These results indicated that NLC–Melox can provide a more controlled and sustained release of Melox compared with the conventional suspension, which releases the drug more rapidly and completely. The in vitro release study of the three types of NLC–Melox-loaded polymeric gels (HPMC, ZnHA, and NaHA) revealed distinct release patterns over 24 h; the release was influenced by their chemical properties and solubility. HPMC exhibited a prolonged and sustained release profile, characterized by an initial burst release of 6.40% at 2 h, and a 27.32% release at 24 h. In contrast, ZnHA showed a slower initial release, with only 1.80% released in the first 2 h, finally reaching up to 44.41% at 24 h, suggesting gradual dissolution and matrix breakdown. NaHA, on the other hand, released 55.07% of Melox in 24 h with an initial rapid release. Comparatively, HPMC provided a more prolonged and controlled release, making it suitable for sustained drug delivery applications, while the rapid release of NaHA may be ideal for quick drug delivery. ZnHA, with its delayed but sustained release, offers a balance between the two release profiles (Figure 10). These findings highlight the importance of selecting the appropriate matrix based on the desired release kinetics of drug delivery systems.

The model-fitting outcomes are outlined in Table 3. Among the four models evaluated, the Korsmeyer–Peppas model demonstrated the highest R^2^ value, where calculated n values indicated the release mechanism for all samples [69].

The power law, also known as the Korsmeyer–Peppas model ($Mt∕M\alpha ={Kt}^{n}$), suggests a diffusion-controlled mechanism; this model was employed to describe and analyze drug release from polymeric nanoparticle dosage forms, such as hydrogels, or in cases, where the release process involves multiple kinetic mechanisms [70]. In the Korsmeyer–Peppas equation, Mt/Mα is the fraction of drug released or diffused as a power function of time (t), while (n) serves as the release exponent to define the underlying drug transport mechanism in matrix systems. When the n value falls within the range of 0.45 to 0.89, it indicates a non-Fickian release pattern, where diffusion acts as the primary mechanism. Nevertheless, the swelling of the matrix stabilizes much quicker than the rate of drug release, so diffusion happens predominantly on the swollen polymer matrix [71]. In the case of n ˃ 0.89, the main release mechanism is super case II transport, predominantly controlled by polymer relaxation and erosion [72], which was present in the case of the NLC–Melox-ZnHA gel.

### 3.10. In Vitro Anti-Inflammatory Study

BSA denaturation assay is an established in vitro method used to evaluate the anti-inflammatory activity of compounds or formulations [73]. The principle underlying this method is based on the denaturation of BSA upon exposure to heat, which results in structural alterations and functionality of the protein. This denaturation process triggers the inflammatory response to counteract the perceived threat. Assessing the ability of potent agents to prevent or suppress this process provides insights into their potential anti-inflammatory efficacy [74].

**Figure 11:**
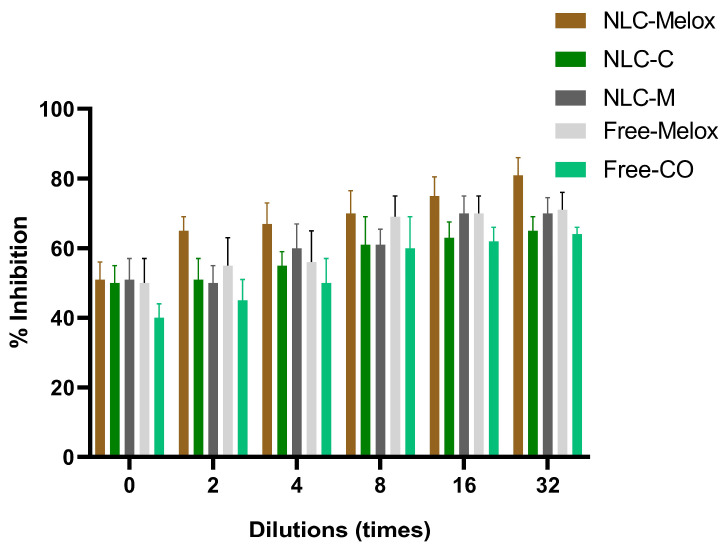
% Denaturation inhibition of BSA with NLC–Melox (containing both Melox and CO), NLC-C (containing only CO), NLC-M (containing only Melox), free Melox, and CO.

The anti-inflammatory potential of NLC–Melox (containing Melox and CO), NLC-M (containing only Melox), and NLC-CO (containing only CO to assess its therapeutic effect) was evaluated by measuring their ability to inhibit heat-induced denaturation of protein. The percentage inhibition of BSA denaturation was evaluated across a range of dilutions, as summarized in Figure 11.

At 0 dilution, all three formulations (NLC–Melox, NLC-C, and NLC-M) exhibited comparable inhibition rates, with values of 51 ± 5%, 50 ± 5%, and 51 ± 6%, respectively. As the concentration decreased at higher dilutions, NLC–Melox exhibited a progressive and significant enhancement in anti-inflammatory activity, reaching a maximum inhibition of 81 ± 5%. In contrast, NLC-C and NLC-M showed a moderate increase in inhibition, i.e., 65 ± 4% and 70 ± 5% at the same dilution. Two-fold dilutions of Melox and CO were tested individually to evaluate their pharmacological activity in free form. The results demonstrate that the anti-inflammatory activity of the free Melox and CO was comparable to that of the encapsulated formulation, indicating that the encapsulation process preserved the therapeutic efficacy of the active compounds.

These results highlighted the superior anti-inflammatory efficacy of the combined ingredients formulation (NLC–Melox), which synergistically leverages the therapeutic properties of both Melox and CO. The findings suggested that NLC–Melox is a promising candidate for further development, offering enhanced protection against protein denaturation at comparatively lower concentrations than formulations containing individual components.

The results outlined above align with the research conducted by Taha et al., who investigated the anti-inflammatory potential and safety profile of Melox in various formulations. In their study, Melox treatments at lower concentrations were established to be non-cytotoxic and safe for use in contrast to higher-dose preparations (up to 50 μg/mL). The anti-inflammatory efficacy of Melox-loaded solid dispersions (SDs) was assessed by measuring the percentage production inhibition of nitric oxide (NO) in lipopolysaccharide (LPS)-stimulated RAW 264.7 macrophages. Results showed that SDs at a concentration of 10 μg/mL of Melox exhibited significant anti-inflammatory activity and achieved up to 63% inhibition of NO production [9].

### 3.11. Antibacterial Study

The antibacterial agar plate study relies on the diffusion of antimicrobial agents from a sample into an agar medium inoculated with bacterial strains. Table 4 summarizes the details of all tested samples against both bacterial strains.

Referring to results in the case of *A. actinomycetemcomitans*, the antibacterial effect stems solely from CO for NLC-C, while NLC-M and free Melox displayed no inhibitory activity against the bacterial strains. The highest antimicrobial activity was observed with the NLC–Melox formulation, indicating a significantly enhanced antibacterial effect compared to free CO.

NLC-ZnHA gel also exhibited consistent and improved activity, with inhibition zones of 30.3 ± 1.3 mm at 24 h and 30.7 ± 0.6 mm at 48 h. In contrast, the NLC-NaHA gel showed moderate activity, with inhibition zones of 24.0 ± 0.0 mm at 24 h and 24.3 ± 1.2 mm at 48 h. For the NLC-HPMC gel, the inhibition zone was 32.3 ± 1.5 mm at 24 h, which decreased to 30.0 ± 0.0 mm at 48 h, suggesting a reduction in activity over time. These results highlighted the varying degrees of antimicrobial efficacy among the formulations, with NLC–Melox standing out as the most effective in the initial 24 h. The consistent performance of NLC-ZnHA gel further underscores its potential as a reliable antimicrobial formulation, while the moderate activity of NLC-NaHA and the decline in the efficacy of the NLC-HPMC gel warrant further investigation to enhance their therapeutic potential.

In the case of the NLC-HPMC gel formulation, the initial effect is more pronounced, which gradually diminishes over time. This can be attributed to the comparatively slow release of the drug from the polymeric system as time progresses. The release of drugs from polymeric hydrogels is influenced by several factors. For instance, the process is regulated by the diffusion of water into the matrix, the dissolution of the drug, and the diffusion of the drug from the system, all of which are affected by interactions between the polymer and the loaded entities. Furthermore, the thickness of the hydrated matrix plays a critical role in determining the diffusional path length of the drug and the rate of its release [75].

Blank HPMC and NaHA gels did not show any antibacterial activity, while ZnHA blank gel demonstrated inherent antibacterial properties, presumably due to the action of zinc ions [76]. The antibacterial efficacy was significantly augmented upon the addition of NLC–Melox, in which a therapeutically active antibacterial oil (CO) was incorporated into the ZnHA gel.

The reduced antibacterial activity of CO observed after NLC’s incorporation into gels can be attributed to the controlled release mechanism facilitated by the gel formulations. Encapsulation within NLCs and subsequent integration into gel matrices significantly control the availability of the active component, which is crucial for their prolonged antimicrobial efficacy. This controlled release behavior mirrors the findings of our in vitro release studies, where the gels demonstrated a more sustained, slower, and prolonged release profile compared with NLCs. The gel matrix, along with the lipid core of the NLCs, likely modulates the diffusion and partitioning of CO and contributes to antibacterial activity. These findings highlight the trade-off between achieving sustained release and maintaining antimicrobial efficacy. The formulations exhibited limited to no antimicrobial activity against *S. mutans*. Specifically, the NLC-ZnHA gel, NLC-NaHA gel, and NLC-HPMC gel showed no detectable inhibition zones throughout the study time span. Blank ZnHA gel exhibited inhibitory activity against *S. mutans*, consistent with the established antimicrobial efficacy of zinc ions against this pathogen, as reported in studies on ZnO nanoparticles [77]. The NLC–Melox formulation exhibited marginal inhibitory efficacy against *S. mutans*, yielding inhibition zones of 14.7 ± 1.0 mm and 14.4 ± 1.1 mm at 24 and 48 h, respectively. Concurrently, CO displayed negligible inhibitory activity against this strain, corroborating prior literature documenting an inhibition zone of 13.0 mm [78]. Collectively, these findings underscore the limited overall efficacy of all the tested formulations in suppressing *S. mutans* growth.

Interestingly, NLC-C did not exhibit a measurable zone of inhibition against *S. mutans*, necessitating MIC determination to accurately assess the effective antibacterial concentration of CO-loaded NLCs. Thus, the MIC assay was conducted to overcome the limitations of the agar diffusion method and to obtain more precise and reliable results.

The MIC and MBC values are presented in Table 5. Between the two bacterial strains, NLC–Melox formulation demonstrated enhanced antibacterial activity against *A. actinomycetemcomitans*, as reflected in both MIC and MBC values (312.5 µg/mL), whereas the NLC-C formulation showed a lower MIC of 156.2 µg/mL but a higher MBC value (625 µg/mL).

CO emulsion showed a four-fold higher MBC (1250 µg/mL) than that of NLC–Melox (312.5 µg/mL). The incorporation of the antibacterial agent into nanoparticles enhanced its activity against the tested bacterial strain. Since both the free drug and the nanoparticle formulations were tested at the same concentrations, the observed increase in antibacterial activity of nanoformulations is likely attributed to their increased adhesion to bacterial surfaces [79] and improved nanoparticle penetration into bacterial cells [80]. This phenomenon could enhance the therapeutic efficacy of the system by facilitating the localized release of drug molecules in proximity to their intended target [81].

The NLC–Melox formulation exhibited higher MIC and MBC values against *S. mutans* than *A. actinomycetemcomitans* with an MBC/MIC ratio of 8. This ratio signifies a bacteriostatic effect of CO, given that values ≤4 are indicative of bactericidal activity, whereas ratios exceeding 4 denote a bacteriostatic effect [82]. Eugenol, the main constituent of CO, markedly reduces the acid production, adherence ability, and water-insoluble glucan synthesis of *S. mutans* [83]. A previous study demonstrated that eugenol inhibited biofilm formation by *S. mutans,* indicating that its mode of action involves the disruption of virulence factors rather than the induction of bactericidal effects [84]. Overall, these findings indicate that nanoparticle-based delivery systems markedly enhance the antibacterial efficacy of the incorporated agent, as reflected by the observed MIC and MBC values.

### 3.12. Stability Study

Stability assessment is primarily conducted to ensure that the formulation maintains its quality, safety, and efficacy throughout its intended shelf life. In the case of NLC–Melox, a negligible variation was observed in the Z-average (186 ± 1.0 nm). The particle distribution remained consistently narrow (0.28 ± 0.5). Additionally, the ZP value (−20 mV) indicates steric stabilization. TEM images (Section 3.2) reveal that gelling systems effectively enhanced the long-term stability of nanoparticles, ensuring their functionality and performance over extended storage periods.

## 4. Conclusions

This study successfully formulated and assessed therapeutic NLCs loaded with Melox in mucoadhesive hydrogels for periodontitis treatment. Our results showed that these nanoformulations significantly improved drug solubility and targeted delivery while ensuring controlled release and extended retention. Among the hydrogels tested, ZnHA demonstrated superior mucoadhesion, structural integrity, and antibacterial properties, establishing it as the most promising option for periodontal therapy. A 3-month stability study confirmed the potential of the formulation for long-term effectiveness and stability in periodontal disease therapy. Additionally, the in vitro anti-inflammatory assay underscored the synergistic efficacy of Melox and CO in modulating inflammation. Notably, the antibacterial activity was solely attributed to CO, with the MIC and MBC values further validating its potency.

Taken together, these findings suggested that NLC-based mucoadhesive gels hold significant potential as a novel strategy for the local treatment of periodontitis. By addressing the key limitations of conventional therapies, such as poor drug retention and rapid clearance, our formulations offered a promising alternative for enhanced therapeutic outcomes. Future work will focus on cell line cytotoxicity and anti-inflammatory studies, along with in vivo evaluation, to further assess the translational potential of this advanced drug delivery system.

## Figures and Tables

**Table 1:** Constituents of all the components of NLC–Melox and NLC-loaded gelling systems.

Formulations	S.L (%)	CO (%)	Surfactant (%)	Melox (μg/mL)	HPMC (%)	ZnHA (%)	NaHA (%)
NLC–Melox	3	2	2.5	250	-	-	-
NLC-HPMC gel	3	2	2.5	250	1	-	-
NLC-ZnHA gel	3	2	2.5	250	-	1	-
NLC-NaHA gel	3	2	2.5	250	-	-	1

**Table 2:** Thermal analysis characteristics of formulations measured by DSC.

Formulations	Enthalpy Change (J/g)	Onset Temperature(°C)	Peak Point (°C)	Endset Temperature (°C)	CI%
NLC-NaHA gel	67.51	61.12	68.03	72.39	22.4
NLC-ZnHA gel	61.05	62.95	68.99	73.36	20.2
NLC-HPMC gel	55.63	60.96	68.98	73.35	18.4
NLC–Melox	49.11	65.57	69.15	70.42	16.3
S.L	100.3	71.49	73.05	74.97	-

**Table 3:** Release kinetic models of NLC–Melox and loaded gels. Data present the fitting of the models (R^2^) and the release exponent of the Korsmeyer–Peppas model.

Kinetic Release Models	NLC–Melox	NLC-HPMC Gel	NLC-ZnHA Gel	NLC-NaHA Gel
Zero order	0.88	0.86	0.97	0.88
First order	0.95	0.90	0.97	0.94
Higuchi	0.96	0.97	0.85	0.96
Korsmeyer–Peppas	0.98	0.99	0.98	0.98
(n)	0.61	0.58	0.95	0.60

**Table 4:** Zone of inhibition presenting antibacterial activity of NLCs and NLC-loaded gels against bacterial strains.

Formulations	Zone of Inhibition (mm)			
	*A. actinomycetemcomitans*		*S. mutans*	
	24 h	48 h	24 h	48 h
NLC-ZnHA gel	30.3 ± 1.3	30.7 ± 0.6	N.A.	N.A
NLC-NaHA gel	24.0 ± 0.0	24.3 ± 1.2	N.A.	N.A.
NLC-HPMC gel	32.3 ± 1.5	30.0 ± 0.0	N.A.	N.A
ZnHA blank gel	25.0 ± 1.0	24.7 ± 1.2	14.5 ± 0.0	14.2 ± 0.3
NaHA blank gel	N.A.	N.A.	N.A.	N.A.
HPMC blank gel	N.A.	N.A.	N.A.	N.A.
NLC–Melox	36.3 ± 2.1	34.3 ± 2.9	14.7 ± 1.0	14.4 ± 1.1
NLC-C	33.3 ± 2.3	33.3 ± 1.2	N.A.	N.A.
Free CO	28.3 ± 2.1	26.3 ± 1.5	17.7 ± 0.6	17.5 ± 0.5
NLC-M	N.A.	N.A.	N.A.	N.A.
Free Melox	N.A.	N.A.	N.A.	N.A.

**Table 5:** In vitro antibacterial activities of free and encapsulated drug against two bacterial strains.

Formulations	*A. actinomycetemcomitans*		*S. mutans*	
	MIC (µg/mL)	MBC (µg/mL)	MIC (µg/mL)	MBC (µg/mL)
NLC–Melox	312.5 *	312.5 *	625 *	5000 *
NLC-C	156.2 *	625 *	625 *	1250 *
CO	312.5 *	1250 *	1250 *	5000 *
NLC-M	>250 **	>250 **	>250 **	>250 **
Melox	>250 **	>250 **	>250 **	>250 **

* concentration of CO. ** concentration of Melox.

## Data Availability

Data are contained within the article.

## Supplementary Materials

The following supporting information can be downloaded at: https://www.mdpi.com/article/10.3390/pharmaceutics17050620/s1, Table S1. NLC composition with DLS characterization. Figure S1. Results of Raman measurements for samples with an increased concentration of Melox. (a) Raman spectra of NLC–Melox, S.L, CO, surfactant, and Melox. The C=O and C=C stretching of Melox in the range of 1600–1400 cm^−1^ is marked with an arrow; (b) Raman spectra of NLC–Melox loaded gels (HPMC, NaHA, and ZnHA) compared with raw polymers, NLC–Melox, and Melox. Characteristic peaks of Melox appeared in all formulations at 1526 cm^−1^, while at 1160 cm^−1^, a comparatively small peak appeared in the case of NLC–Melox and HPMC gel, and a weak signal appeared in hyaluronate-based gel systems. Raman spectroscopy confirmed the structural integrity of Melox post-encapsulation, with characteristic peaks remaining unaltered.
