# Supplementary material for: Hydrogel–Nanolipid Formulations for the Complex Anti-Inflammatory and Antimicrobial Therapy of Periodontitis
Source: Pharmaceutics. 2025 May 7;17(5):620. doi: 10.3390/pharmaceutics17050620 (PMC12114638; doi:10.3390/pharmaceutics17050620)
Supplement: Supplementary file 1 [file pharmaceutics-17-00620-s001.zip › pharmaceutics-3578040-supplementary.pdf]

2.2.1. Preliminary Study

Table S1. NLC composition with DLS characterization.

Formulations	Composition			DLS results		
	S.L (%)	CO (%)	Surfactant (%)	Z-average (nm)	PDI	ZP (mV)
A	3.0	2.0	2.5	183.0	0.26	-19.5
B	3.0	2.0	4.0	184.0	0.26	-16.3
C	3.5	1.5	5.0	27.7	0.22	-16.3
D	3.0	2.0	5.0	51.6	0.45	-16.6
E	4.0	1.0	5.0	100.0	0.39	-10.7
F	2.5	2.5	5.0	67.8	0.50	-12.9

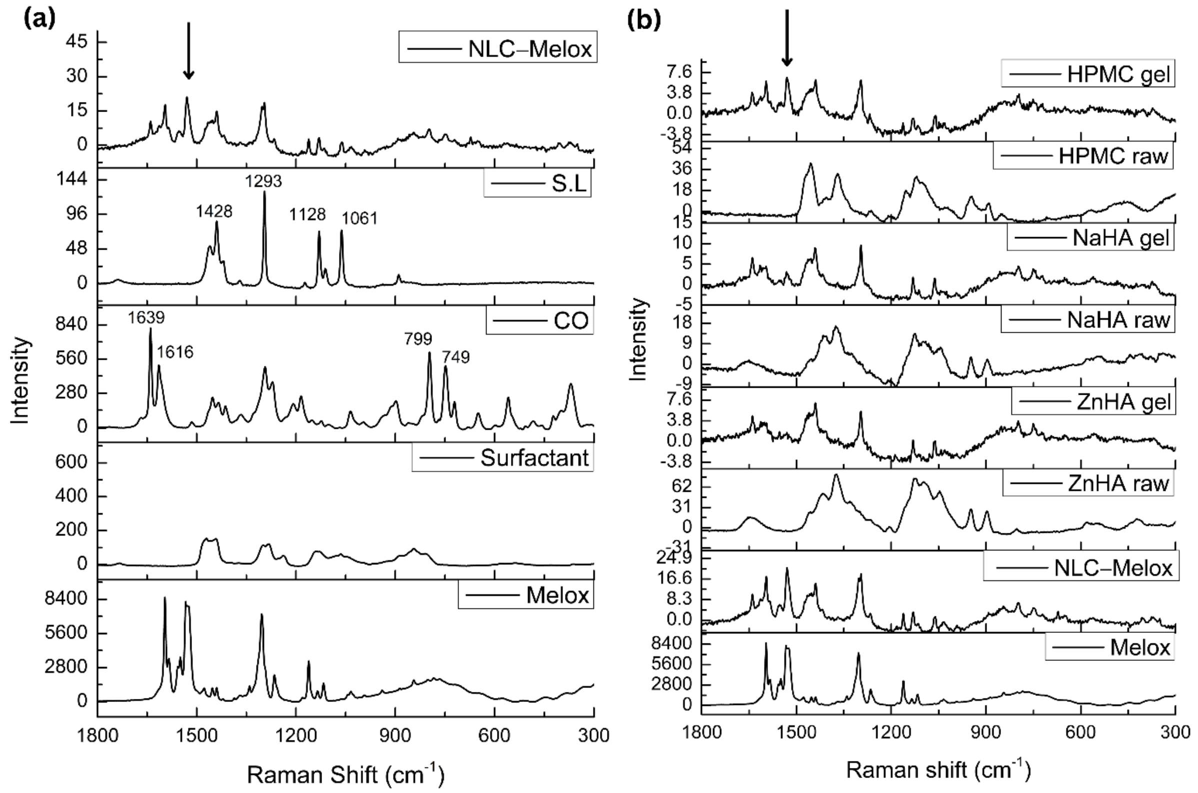

Figure S1. Results of Raman measurements for samples with an increased concentration of Melox. (a) Raman spectra of NLC–Melox, S.L, CO, surfactant, and Melox. The C=O and C=C stretching of Melox in the range of 1600–1400 cm^{-1} is marked with an arrow; (b) Raman spectra of NLC–Melox loaded gels (HPMC, NaHA, and ZnHA) compared with raw polymers, NLC–Melox, and Melox. Characteristic peaks of Melox appeared in all formulations at 1526 cm^{-1}, while at 1160 cm^{-1}, a comparatively small peak appeared in the case of NLC–Melox and HPMC gel, and a weak signal appeared in hyaluronate-based gel systems. Raman spectroscopy confirmed the structural integrity of Melox post-encapsulation, with characteristic peaks remaining unaltered.
